# Asymtomatic Bacteriuria as a Model to Study the Coevolution of Hosts and Bacteria

**DOI:** 10.3390/pathogens5010021

**Published:** 2016-02-15

**Authors:** Ulrich Dobrindt, Björn Wullt, Catharina Svanborg

**Affiliations:** 1 Institute of Hygiene, University of Münster, Mendelstr. 7, D-48149 Münster, Germany; 2 Department of Microbiology, Immunology and Glycobiology, Institute of Laboratory Medicine, Lund University, Sölvegatan 23, S-223 62 Lund, Sweden; bjorn.wullt@med.lu.se (B.W.); catharina.svanborg@med.lu.se (C.S.)

**Keywords:** asymptomatic bacteriuria (ABU), *E. coli* adaptation, evolution

## Abstract

During asymptomatic bacteriuria (ABU), bacteria colonize the urinary tract for extended periods of time without causing symptoms of urinary tract infection. Previous studies indicate that many *Escherichia coli* (*E. coli*) strains that cause ABU have evolved from uropathogenic *E. coli* (UPEC) by reductive evolution and loss of the ability to express functional virulence factors. For instance, the prototype ABU strain 83972 has a smaller genome than UPEC strains with deletions or point mutations in several virulence genes. To understand the mechanisms of bacterial adaptation and to find out whether the bacteria adapt in a host-specific manner, we compared the complete genome sequences of consecutive reisolates of ABU strain 83972 from different inoculated individuals and compared them with the genome of the parent strain. Reisolates from different hosts exhibited individual patterns of genomic alterations. Non-synonymous SNPs predominantly occurred in coding regions and often affected the amino acid sequence of proteins with global or pleiotropic regulatory function. These gene products are involved in different bacterial stress protection strategies, and metabolic and signaling pathways. Our data indicate that adaptation of *E. coli* 83972 to prolonged growth in the urinary tract involves responses to specific growth conditions and stresses present in the individual hosts. Accordingly, modulation of gene expression required for survival and growth under stress conditions seems to be most critical for long-term growth of *E. coli* 83972 in the urinary tract.

## 1. Introduction

Normal flora furnishes the host with ecological barriers that prevent pathogen attack while maintaining tissue homeostasis. Despite their vast numbers and staggering molecular complexity, microbiomes of the gut, respiratory and urogenital tracts persist without triggering a destructive host response. This lack of destructive inflammation is fascinating and important, as it reflects exquisite molecular regulation of the host environment by commensal bacteria. Simultaneously, the host regulates permitted and unwanted paths of immune activation by discriminating attacking pathogens from beneficial commensals.

The failure of asymptomatic carrier strains to trigger disease-associated signaling pathways and pathology has generally been attributed to their lack of virulence and, until recently, it was not clear if, in addition, asymptomatic carrier strains enhance their persistence by actively modifying the host environment. We have discovered that commensal bacteria modulate host gene expression to ensure that destructive immune activation will not occur [1]. These immune-modulatory mechanisms provide a rich source for novel therapeutic interventions against pathogen-specific, disease-associated host responses, which are not evoked by closely related non-pathogenic variants. This is especially important since a paradigm shift is needed to minimize the use of antibiotics and to develop new, appropriate therapies.

## 2. Urinary Tract Infection (UTI)

UTI constitutes a highly relevant model of microbial adaptation, in which the contrasting roles of pathogens and commensals are clearly displayed [2]. Pathogens disrupt the mucosal barrier in the kidneys, causing severe, potentially life-threatening disease, urosepsis and mortality. Recurrent, acute pyelonephritis is also a cause of end-stage renal disease, with the associated morbidity and need for dialysis and transplantation, in addition to the personal and economic burdens associated with chronic illness.

Innate immunity controls the resistance to UTI. Uropathogenic *Escherichia coli* (UPEC) activate an innate immune response through virulence factor–specific TLR4 signaling, the TRIF/TRAM adaptors, MAPK-, p38- and CREB phosphorylation and IRF3/IRF7, AP1-dependent transcription. We have shown that *Irf3^−/−^* mice develop severe, acute symptoms accompanied by urosepsis and renal abscess formation, demonstrating that the innate immune response orchestrated by IRF-3 is crucial for bacterial clearance and for renal tissue integrity. Human disease relevance is suggested by an increased frequency of functionally relevant *IRF3* promoter polymorphisms in about 70% of patients with recurrent acute pyelonephritis [3].

Recently, the soluble pattern recognition molecule pentraxin 3 (PTX3) was identified as a novel determinant of host resistance to UTI [4]. PTX3 is a key component of the humoral arm of innate immunity and Ptx3-deficient mice showed defective control of UTI, increased tissue inflammation and tissue damage. PTX3 was detected in patient urine and PTX3 polymorphisms correlated with APN susceptibility, identifying PTX3 as the first humoral pattern recognition molecule in innate resistance against UTI.

## 3. Asymptomatic Bacteriuria (ABU) Is Protective

ABU is the most common form of UTI and the majority of ABU cases are caused by *E. coli*. Asymptomatic bacterial carriage in the bladder resembles commensalism at other mucosal sites. While a variety of bacteria may establish ABU [5], the identification of species other than *E. coli* should be regarded with caution as a potential sign of impaired host defense functions and problems associated with multi-drug resistance. Patients with ABU may carry the same strain for months or years without developing a disease response, leaving these commensal-like bacteria to successfully co-evolve with their hosts in a niche with little microbial competition. In epidemiological studies, ABU has been shown to protect against recurrent, symptomatic infection with more virulent strains [6].

**Figure 1:**
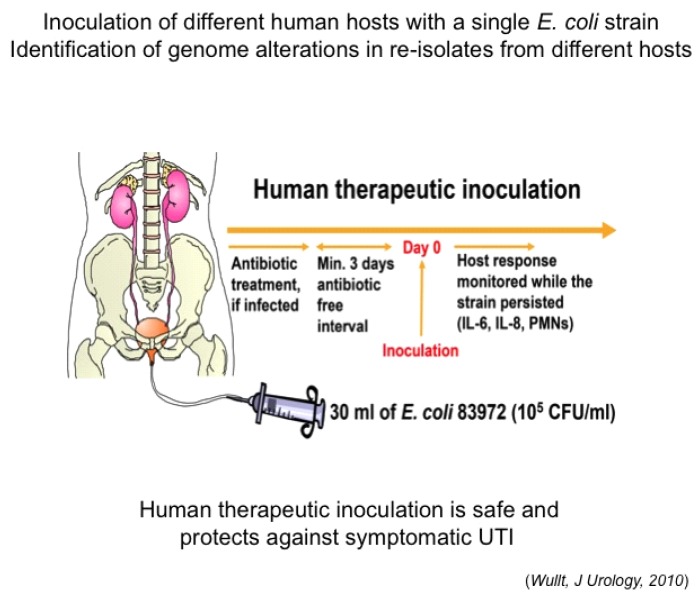
Deliberate bladder inoculation with *E. coli* 83972 is used therapeutically, to protect against recurrent urinary tract infection.

We have used this protective effect as a rationale to deliberately establish ABU in patients with therapy-resistant recurrent UTI (Figure 1) [7]. The prototype ABU strain *E. coli* 83972 was first isolated during an epidemiologic study of ABU in schoolgirls [6] and was selected for human inoculation as it fails to express virulence factors associated with symptomatic UTI and lacks conjugative plasmids [8]. Therapeutic urinary tract inoculation with *E. coli* 83972 establishes persistent, protective bacteriuria, as demonstrated in placebo-controlled studies [7,9]. Clinical use of *E. coli* 83972 has recently been endorsed in the European Urology Guidelines from 2014.

## 4. Results and Discussion

### 4.1. Bacterial Evolution towards Commensalism rather than Virulence

Bacteria increase their fitness in new host niches by rapid adaptation to changing environmental conditions. They lose or gain genetic material and, through selection, new variants become fixed in the population. Evolution has mainly been assumed to favor virulence, which promotes host-to-host spread and tissue attack. Until recently, evolution of commensalism had not been considered. We have proposed that ABU strains evolve towards commensalism in human hosts, as defined by a reduction in overall genome size, inactivation of virulence genes and modifications of transcriptional regulators [10,11]. Some ABU strains, such as the ABU *E. coli* strain VR50, have been also shown to evolve from commensal strains by gaining colonization factors [12].

**Figure 2:**
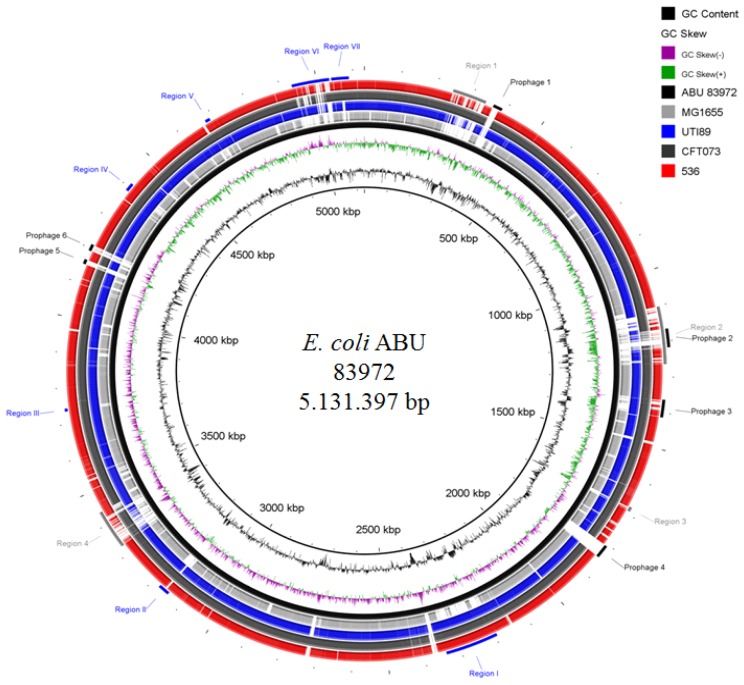
Comparative genome sequence analysis of ABU *E. coli* isolate 83972. Comparison of the chromosome of *E. coli* strain 83972 (ABU isolate), *E. coli* K-12 strain MG1655, and *E. coli* UTI isolates UTI89, CFT073 and 536. The localization of rather strain-specific or conserved genomic islands (regions 1–4 and regions I–VII) as well as of prophage genomes (prophage 1–6) in the *E. coli* 83972 chromosome is indicated.

Epidemiologic studies have established that the severity of UTI reflects the virulence profile of the infecting strain, with a higher frequency of tissue-attacking virulence factors expressed by UPEC strains than by most strains causing ABU [13,14], despite the presence of virulence gene sequences in many ABU strains [15]. Until recently, the molecular basis for this discrepancy has not been examined. Our results have established that ABU strains evolve towards commensalism through reductive evolution in human hosts [10], resulting in overall genome size reduction and systematic inactivation of virulence genes, either by the accumulation of point mutations or deletions [11,16]: Sequencing of the *E. coli* 83972 genome revealed a common ancestry with uropathogenic *E. coli* strains but a smaller genome size due to multiple deletions and mutations, suggesting that this strain adapted to the human urinary tract by undergoing reductive evolution [10,16] (Figure 2). For example, a large *fim* deletion and several *papG* point mutations abolish fimbrial expression and adherence [11]. Extended sequencing and phenotypic characterization has since confirmed that ABU strains undergo reductive evolution by a reduction in genome size and an accumulation of genomic alterations, which result in specific loss of expression or decay of UPEC virulence genes [17].

### 4.2. Host-Specific Genome Alterations in Inoculated Hosts

To determine adaptation during long-term bladder colonization, we analyzed the genome sequence of ABU *E. coli* isolate 83972 (Figure 2) and compared this sequence to genomes of other model UPEC strains and non-pathogenic *E. coli* K-12 strain MG1655 to identify strain-specific genomic regions. Re-sequencing of *E. coli* 83972 reisolates after therapeutic bladder colonization of different patients led to the observation that hosts personalize their bacteria, thus providing the first, genome-wide example of a single bacterial strain’s evolution in different, deliberately inoculated patients. A general survey of the genome structure of *E. coli* 83972 reisolates from six patients revealed marked differences between patient reisolates and the ancestral strain [10]. We identified 34 mutations, which affected metabolic and virulence-related genes. Frequently, genes coding for regulators with pleiotropic function were affected. The comparative genomic analysis showed that the individual number of mutations within the genomes of *in vivo* reisolates was markedly higher than in *in vitro*–evolved *E. coli* 83972 descendants.

**Figure 3:**
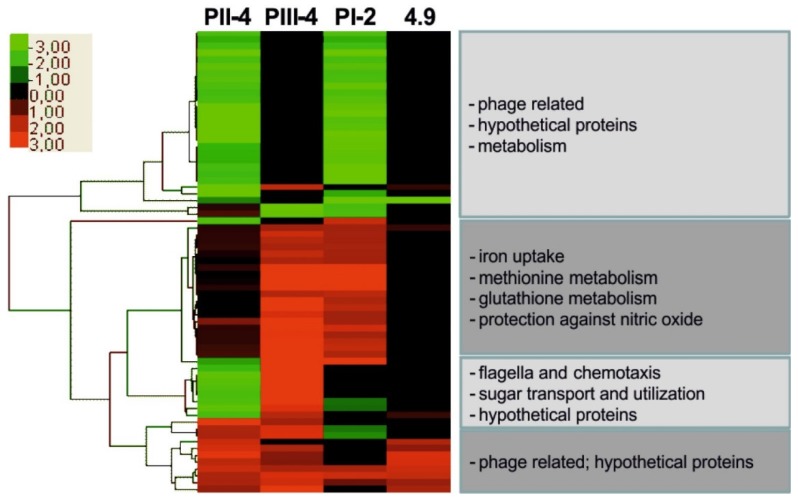
Gene expression in reisolates of *E. coli* 83972 from individual hosts. Hierarchical clustering of deregulated genes in *in vivo* reisolates PI-2, PII-4 and PIII-4 and *in vitro* grown strain 4.9 relative to parent *E. coli* strain 83972 upon *in vitro* growth in pooled human urine [10].

Several genomic loci were independently affected in different individual patient reisolates, but not upon prolonged propagation in the *in vitro* evolution experiment. These loci may represent mutation hotspots under positive selection *in vivo*, indicating that *E. coli* is subjected to conserved evolutionary patterns during prolonged bladder colonization. During colonization of the urinary tract, bacteria are exposed to high levels of reactive oxygen and/or nitrogen species, which are either produced to support host defense mechanisms or which can be generated during anaerobic bacterial growth in urine. Due to the presence of inorganic ions and urea, urine imposes marked osmotic stress on bacterial cells. The independent acquisition of mutations in genes involved in oxidative or osmotic stress response supports the idea that adaptive evolution of corresponding traits improves bacterial fitness in this niche. A marked difference between the reisolates was also demonstrated by transcriptome analysis (Figure 3). Overall, the affected genes are involved in osmoregulation, in oxidative stress response and in global regulation of virulence and fitness traits. The analysis of consecutive reisolates from the same patient confirmed that several genomic alterations were present in the early and in the later reisolates, suggesting that growth in the host environment requires and promotes the stabilization of these genomic changes. Thus, our results provide evidence that adaptive bacterial evolution is driven by individual host environments.

This host-specific loss of gene function supports the hypothesis that evolution towards commensalism rather than virulence is favored during asymptomatic bladder colonization. Ongoing collaborative studies aim to identify the mechanisms by which different hosts personalize their microbiota at the genomic level and the host response variables that drive bacterial adaptation by continuous monitoring of host transcription in individual patients, as well as their state of health and proteomic profile, including inflammatory mediators and immune variables. We also observed that ABU strains actively modify the host environment by inhibiting RNA polymerase II–dependent gene expression [1] and Ambite *et al.*, this issue. The molecular determinants of evolution towards commensalism and host modulation remain to be defined. Innate immune activation is limited in ABU [18], suggesting that different host response pathways and effector molecules drive bacterial commensalism. Human therapeutic inoculation offers a unique setting to study the *in vivo* interactions between commensals and their human hosts in sufficient detail to address those questions.

### 4.3. Host-Specific Gene Expression Levels and Phenotypic Variation in Inoculated Hosts

**Figure 4:**
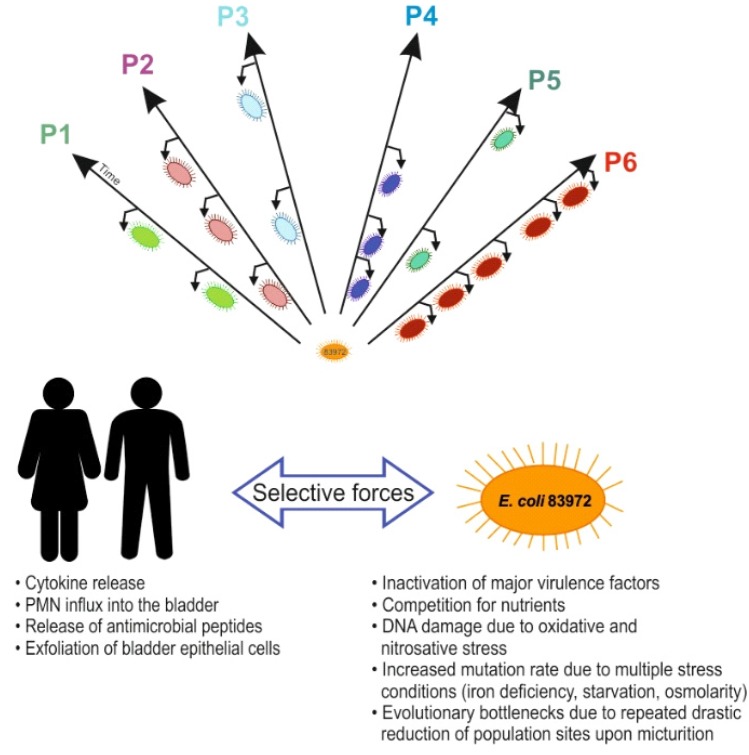
Factors affecting genome plasticity and host-dependent adaptation of *E. coli* in the urinary tract. Deliberate colonization of individual patients (e.g., P1–P6) with *E. coli* strain 83972 allows studying host-dependent adaptation of *E. coli* strain 83972 over prolonged periods of time. Colonization of the urinary tract of individual patients results in various selective pressures due to individual host responses, nutrient supply and bacterial competition that drive host-specific bacterial adaptation.

Diverse pheno- and genotypic comparisons uncovered striking differences among *in vivo* 83972 reisolates, which were rarely observed in *in vitro*–evolved *E. coli* 83972 reisolates. To evaluate changes on the transcriptional level, transcriptome analyses were performed and the transcriptome of parent strain 83972 was compared with those of selected *in vivo* and *in vitro* reisolates upon growth in pooled human urine. The number of significantly deregulated genes was, on average, four-fold higher than in the *in vivo*–evolved strains compared with the *in vitro*–grown strain. These genes were mainly involved in different stress responses, iron acquisition, metabolic versatility and LPS biosynthesis, but the expression patterns differed between reisolates from different patients [10] (Figure 4). *In vivo* reisolates of *E. coli* strain 83972 differed in growth characteristics (e.g., growth rate and competitive fitness in pooled human urine). Reisolates with slower growth rates in urine were also less competitive relative to ancestor strain 83972. Many reisolates formed less biofilm than parent strain 83972. This phenotype could be correlated with motility and flagella expression [10]. Accordingly, the expression of virulence or fitness traits in *E. coli* is modulated in response to the individual host. Further genome-wide, transcriptome and proteome analysis proved that these genomic changes altered bacterial gene expression, resulting in unique adaptation patterns in each patient affecting iron uptake strategies as well as protection against oxidative or nitrosative stress, general stress response, and utilization of different carbon sources [10,19].

A number of different microbial strategies for bacterial persistence in urine have been discussed (for a recent review see [20]). So far, it remains unclear to what extent changes in bacterial growth rate influence the adaptation in individual hosts. Persistence in the urinary tract might be affected by alterations in bacterial growth rates as metabolic responses of the bacteria are influenced by nutrient availability, general urine composition, presence of antimicrobial factors in urine and by the host response. In addition, *E. coli* strains have specific strategies for protection against osmotic stress, and for resisting the bactericidal effects of the host defenses.

Interestingly, we observed phenotypic variation in the *E. coli* 83972 monoculture populating the bladder [19]. This phenotype switching mirrors adverse and stress conditions, and it may ensure the fitness and survival of a subset of cells in this niche. The occurrence of two phenotypes in a clonal population [20] suggests bistable gene expression, which can be used by the bacteria to efficiently exploit dynamic host environments and to promote gene expression changes, for example during chronic infection [21]. Small colony variant formation has been correlated with chronic infection and it is considered a survival strategy relying on stress-fit individuals in a heterogeneous population [22]. Therefore, bacterial adaptation to long-term *in vivo* growth in the urinary tract could include phenotype switching. Alternatively, the occurrence of heterogeneous populations at symptomatic episodes may represent spontaneous stochastic events including minor transient populations [23].

*E. coli* 83972 colonizes the human urinary tract without inducing a strong immune response and can even actively suppress host gene expression. Genome comparison demonstrated that this strain is a deconstructed, attenuated uropathogen. Although we were able to shed some light on bacterial strategies and conditions which may promote attenuation and *E. coli* adaptation during prolonged colonization of the urinary tract, specific genomic features of strain 83972, which may account for all aspects of the asymptomatic bladder colonization, have not yet been identified. The detailed analysis of the molecular mechanisms required for the specific bacterium-host interaction resulting in a weak host response will be an important task for future studies to understand how this strain actively modifies the host environment in order to promote persistence.
